# Phytol and its metabolites phytanic and pristanic acids for risk of cancer: current evidence and future directions

**DOI:** 10.1097/CEJ.0000000000000534

**Published:** 2020-01-28

**Authors:** Gerd Bobe, Zhenzhen Zhang, Ryan Kopp, Mark Garzotto, Jackilen Shannon, Yumie Takata

**Affiliations:** a Linus Pauling Institute, Oregon State University, Corvallis; b Department of Animal and Rangeland Sciences, College of Agricultural Sciences, Oregon State University, Corvallis; c Division of Hematology/Oncology, Knight Cancer Institute, Oregon Health & Science University, Portland; d Department of Urology, Portland Veterans Affairs Medical Center, Oregon Health & Science University, Portland; e Department of Urology, Oregon Health & Science University, Portland; f OHSU-PSU School of Public Health, Oregon Health & Science University, Portland; g College of Public Health and Human Sciences, School of Biological and Population Health Sciences, Oregon State University, Corvallis, Oregon, USA

**Keywords:** cancer, phytanic acid, phytanic acid, phytol

## Abstract

This review summarizes the current evidence on the potential role of phytol, a microbial metabolite of chlorophyl A, and its metabolites, phytanic and pristanic acids, in carcinogenesis. Primary food sources in Western diets are the nut skin for phytol and lipids in dairy, beef and fish for its metabolites. Phytol and its metabolites gained interest as dietary compounds for cancer prevention because, as natural ligands of peroxisome proliferator-activated receptor-α and -γ and retinoid X receptor, phytol and its metabolites have provided some evidence in cell culture studies and limited evidence in animal models of anti-carcinogenic, anti-inflammatory and anti-metabolic-syndrome properties at physiological concentrations. However, there may be a narrow range of efficacy, because phytol and its metabolites at supra-physiological concentrations can cause *in vitro* cytotoxicity in non-cancer cells and can cause morbidity and mortality in animal models. In human studies, evidence for a role of phytol and its metabolites in cancer prevention is currently limited and inconclusive. In short, phytol and its metabolites are potential dietary compounds for cancer prevention, assuming the challenges in preventing cytotoxicity in non-cancer cells and animal models and understanding phytol metabolism can be mitigated.

## Introduction

Cancer is the second leading cause of deaths with an estimated 609 604 cancer deaths in 2018 in the US (Siegel *et al.* 2018). Dietary phytol, a diterpene alcohol and microbial metabolite of chlorophyl A, and its poly-methylated branched-chain fatty acid metabolites, phytanic acid (PA; 3,7,11,15-tetramethylhexadecanoic acid) and pristanic acid (PRA; 2,6,10,14-tetramethylpentadecanoic acid), have garnered the interest of cancer prevention researchers due to their ability to serve as natural ligands for peroxisome proliferator-activated receptor (PPAR)-α, PPAR-γ and retinoid X receptor (RXR) (Roca-Saavedra *et al.*, 2017). These receptors are transcription factors leading to downstream effects on metabolism, proliferation and apoptosis (Roca-Saavedra *et al.*, 2017). Furthermore, they upregulate the expression of α-methylacyl-CoA racemase (AMACR), especially in prostate cancer tissue (Verhoeven and Jakobs, 2001). Besides being involved in PA catabolism, AMACR is also critical in the degradation of bile acids, ibuprofen and other methylated fatty acids (Lloyd *et al.*, 2008). Hence, phytol, PA and PRA may be linked to carcinogenesis through involvement in peroxisomal and mitochondrial functions, oxidative stress, inflammatory pathways, cell signal transduction, glucose/energy metabolism and microbial effects.

The Western diet contains about 10 mg/day of phytol (Steinberg, 1989; Brown *et al.*, 1993; Vetter and Schroder, 2010; Roca-Saavedra *et al.*, 2017), which mostly comes from the skin of nuts (Steinberg *et al.*, 1967), 50–100 mg/day of PA and 10–30 mg/day of PRA, which are primarily from lipids in dairy, beef and fish (Steinberg, 1989; Brown *et al.*, 1993; Vetter and Schroder, 2010; Roca-Saavedra *et al.*, 2017). The average circulating PA concentration is below 10 µM, and according to diagnostic criteria for PA-related diseases, the PA concentration below 30 µM is considered within the normal physiological range (Baldwin *et al.*, 2010; Lloyd *et al.*, 2013). Tissue PA concentrations are below 0.3% of total fatty acids (Kataria *et al.*, 2015). Both concentrations are sufficient to elicit chemo-preventive properties in cell culture studies, which are summarized in this review.

Current epidemiological evidence about dietary PA intake or circulating PA concentrations is limited to prostate cancer and non-Hodgkin’s Lymphoma (NHL) with varied results. Out of five studies (Xu *et al.*, 2005; Price *et al.*, 2010; Wright *et al.*, 2012; Ollberding *et al.*, 2013; Wright *et al.*, 2014), dietary intake or circulating concentration of PA or PRA have been linked to increased cancer risk overall or in subgroups in four studies and no association was observed in one study. Major food sources of phytol and its metabolites have been implicated in the etiology of cancer. The World Cancer Research Fund/American Institute for Cancer Research expert panel review (World Cancer Research Fund and American Institute for Cancer Research, 2018) reported strong evidence for a positive association of consuming red meats (including beef) and processed meats with colorectal cancer risk and an inverse association between dairy products and colorectal cancer risk. Further, they reported strong evidence for a positive association between salted fish intake and nasopharyngeal cancer risk. For the other major food sources of phytol and its metabolites, current evidence is insufficient or inconsistent to draw conclusions.

This article reviews the current evidence from cell culture studies, animal feeding studies and human intervention and observational studies, regarding phytol and its metabolites as potential dietary compounds for cancer prevention. Furthermore, this review recommends potential future research directions, which, if proven successful, will allow us to move forward with formulating prevention strategies involving phytol and its metabolites in order to decrease the public health burden of cancer. The goal of this review is to raise awareness of phytol and its metabolites and to foster future research, as outlined in this review.

### Metabolism and physiological concentrations of phytol and its metabolites

**Fig. 1:**
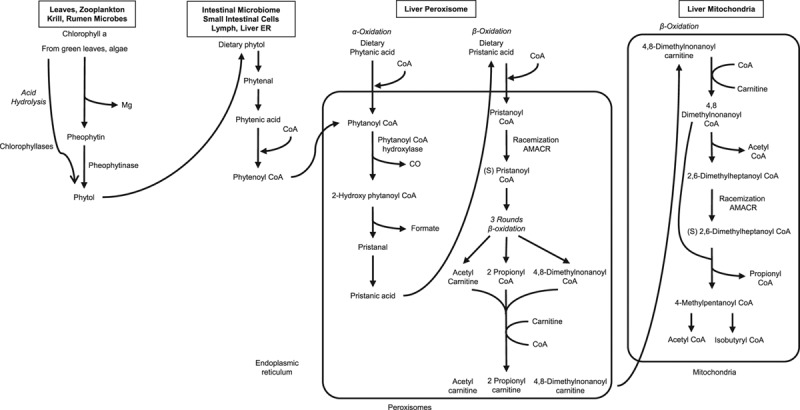
Metabolism of phytol, phytanic acid and pristanic acid. Phytol is derived from plant and phytoplankton chlorophyll. Rumen microbiota, marine zooplankton and human intestinal bacteria are able to cleave the chlorophyll porphyrin ring and release phytol. After intestinal absorption, dietary phytol is metabolized in the endoplasmic reticulum (ER) via phytenal and phytenic acid to phytanoyl CoA. Dietary PA and PRA are activated on the cytosolic side of the peroxisomes to phytanoyl CoA and pristanoyl CoA. In the peroxisomes, phytanoyl CoA is α-oxidized to PRA, the racemic mixture of PRA is converted by α-methylacyl-CoA racemase (AMACR) to (2S) PRA. After three rounds of β-oxidation, 4,8-dimethylnonanoyl CoA is shuttled by carnitine into the mitochondria for complete β-oxidation, which include four rounds of β-oxidation and one racemization step using AMACR (Verhoeven and Jakobs, 2001). PA, phytanic acid; PRA, pristanic acid.

The metabolism of phytol and its metabolites is shown in Fig. 1. Free phytol occurs at low concentrations in plant and animal tissues except for the skin of nuts and plant leaves, where it accumulates and acts as an antimicrobial agent (Steinberg *et al.*, 1967; Islam *et al.*, 2015), suggesting a potential role of phytol in altering the microbiome population. Phytol is derived from plant and phytoplankton chlorophyl. Nearly all phytol in plants is in the bound form as side-chain of chlorophyl or pheophytin and requires microbial enzymes to be released as free phytol (Hansen, 1980). Chlorophyl or pheophytin cannot be absorbed by humans, whereas PA has an absorption rate of 80% (Baxter, 1968). The free phytol is readily converted to PA in the gastrointestinal tract and lymph (Baxter, 1968). As phytol is not produced endogenously (Steinberg *et al.*, 1967), circulating phytol concentrations are probably found at trace amounts (Hansen, 1980). PRA in circulation is derived primarily from endogenous conversion of PA in the liver and a smaller portion from the diet. To our knowledge, the role of phytol and its metabolites on the microbiome is currently unknown and warrants research.

However, at supra-physiological levels, phytol can accumulate in the liver after conversion to PA (Hansen, 1980; Gloerich *et al.*, 2007). Circulating PA is derived primarily from the diet and a small portion (<20%) from endogenous conversion of phytol into PA (Hansen, 1980). As shown in Fig. 1, in the liver, PA is activated to phytanoyl CoA and then transported in the peroxisomes, where phytanoyl CoA is α-oxidized to PRA using various enzymes (Verhoeven and Jakobs, 2001). Mutations in enzymes involved in this step, such as phytanoyl CoA hydroxylase, can result in accumulation of PA as in Refsum disease, but PRA does not accumulate due to such mutations (Hansen, 1980; Verhoeven and Jakobs, 2001). As poly-methylated branched-chain fatty acids, PA and PRA replace primarily *n-3* or *n-6* polyunsaturated fatty acids in tissues (Hansen, 1965; Skrbic and Cumings, 1969; Malmendier *et al.*, 1974). The only reported human tissue concentration of PA is 0.30 wt% of total fatty acids in the prostate tissue (Kataria *et al.*, 2015), but no other tissues. In human populations, serum and plasma PA concentrations were on average under 10 µM [2.6 µM in the EPIC study (Price *et al.*, 2010), 2.9 µM in the North Carolina study (Xu *et al.*, 2005) and 9.8 µM in the Alpha-Tocopherol Beta-Carotene (ATBC) study (Wright *et al.*, 2014)] and differed among meat consumers, vegetarians and vegans (5.77, 3.93 and 0.86 μM, respectively) (Allen *et al.*, 2008), indicating that intake of animal products can increase circulating PA concentrations.

In short, our knowledge about the physiological processes involved in the control of PA and PRA concentrations in absorption, circulation and tissue distribution as well as the effect of the intestinal microbiome on circulating PA and PRA concentrations is limited and requires further research.

### Human consumption patterns of phytol and its metabolites

The current knowledge on phytol and PRA contents in foods is limited (Steinberg *et al.*, 1967; Coppack *et al.*, 1988; Steinberg, 1989; Brown *et al.*, 1993). Major food sources of phytol are the skin of nuts (about 1 g/100 g of food), whereas concentrations in other foods were below 15 mg/100 g of food (Steinberg *et al.*, 1967; Brown *et al.*, 1993). Major food sources of PA are lipids from dairy, beef and fish. Foods with the highest PA content are butter, followed by salmon, halibut and fatty cheeses (Roca-Saavedra *et al.*, 2017). Besides foods, 1 g of fish-oil supplements contain about 10% of the total dietary PA intake. In two previous epidemiological studies, the primary dietary sources of PA were cheese, butter and milk, which in total accounted for up to 53% in the Nebraska Lymphoma Study (NLS) (Ollberding *et al.*, 2013) and 90% in ATBC cohort (Wright *et al.*, 2012). The correlation between circulating PA concentration and fat intake from butter, a major food source of PA, is modest (*r* = 0.35–0.44) (Price *et al.*, 2010; Wright *et al.*, 2014; Kataria *et al.*, 2015). Hence, circulating PA concentrations may serve as dietary biomarker of ruminant fat intake in populations with low fatty fish consumption.

An average Western diet with dairy products contains between 50 and 100 mg/day of PA (Roca-Saavedra *et al.*, 2017) and is estimated to contain about 10–30 mg/day and 10 mg/day for PRA and phytol, respectively, based on the ratio of PA to PRA in dairy products (Steinberg, 1989; Brown *et al.*, 1993; Vetter and Schroder, 2010). This level is similar to the largest feeding study (tested 156 vs. 78 mg/day of PA) (Werner *et al.*, 2013). A few observational studies also reported an estimated intake of PA. In the NLS, the middle tertile of PA ranged from 43.0 to 63.8 mg/day (Ollberding *et al.*, 2013). In the ATBC Cancer Prevention cohort of Finnish male smokers, the average intake in the lowest and highest quartiles of PA were 64 and 199 mg/day, respectively (Wright *et al.*, 2012). Hence, a relatively wide range of intake was reported in healthy populations, although the direct comparison of estimated intake amounts from different studies needs a caution as the studies used different approaches (e.g. the number of food items included in their food frequency questionnaires and statistical methods used to estimate the intake).

### Role of phytol and its metabolites in carcinogenesis

#### Cell culture studies

Phytol and its metabolites at concentrations in the physiological range (≤10 μM) can alter pathways involved in carcinogenesis, such as increasing apoptosis and decreasing proliferation (Komiya *et al.*, 1999; Kim *et al.*, 2015; Thakor *et al.*, 2017). Proposed mechanisms by which phytol and its metabolites exert their chemo-preventive effects include inducing mitochondrial dysfunction, oxidative damage and intracellular Ca^2+^ deregulation, as well as epigenetic changes such as histone deacetylation (Idel *et al.*, 2002; Schönfeld *et al.*, 2004; Schönfeld *et al.*, 2006; Schönfeld and Wojtczak, 2007; Leipnitz *et al.*, 2010; Kruska and Reiser, 2011; Grings *et al.*, 2012; Borges *et al.*, 2015; Dhaunsi *et al.*, 2016; Dhaunsi *et al.*, 2017) (Table 1). The concentrations that resulted in these changes varied from 1 µM of PA for inducing oxidative damage in rat heart cells (Schönfeld *et al.*, 2004) to 100 µM of PA for causing nitric oxide-dependent apoptosis in vascular cells (Schluter *et al.*, 2002). In human embryonic kidney cells (HEK293), 50 μM of PA increased intracellular release of Ca^2+^ reserves, which was mediated via activation of free fatty acid receptor, GPR40 (Kruska and Reiser, 2011). Thus, PA may alter various pathways in different cell types in a dose-dependent manner. Studies of brain tissues showed effects on membrane depolarization, oxidative damage, mitochondrial reactive oxygen species (ROS) generation and histone deacetylation (epigenetic transcription regulation) at concentrations as low as 80 nM of PA (Schönfeld *et al.*, 2004; Reiser *et al.*, 2006; Schönfeld *et al.*, 2006; Schönfeld and Reiser, 2006; Rönicke *et al.*, 2009; Leipnitz *et al.*, 2010; Borges *et al.*, 2015; Nagai, 2015). In HepG2 cells, 40 µM of phytol can suppress the epithelial-mesenchymal transition signaling, which is important for tumor invasion (Kim *et al.*, 2015). Conversely, phytol and its metabolites may also have tumor-promoting properties, as ROS generation can induce DNA damage that either causes apoptosis or transforms normal cells into cancerous cells (Gu *et al.*, 2018; Samimi *et al.*, 2018). Moreover, 0.3 µM of PA or PRA in normal prostate cells was sufficient to increase the protein content of AMACR (Mobley *et al.*, 2003), an enzyme known to be elevated in prostate cancer tissue (Thornburg *et al.*, 2006). Thus, PA may be both friend and foe for cancer prevention and treatment, which warrant further investigation.

**Table 1:**
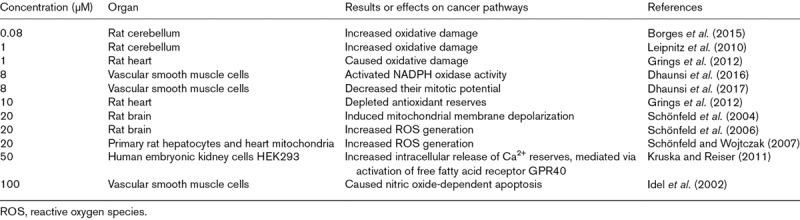
Effect of phytanic acid on cancer pathways in cell culture studies

In the physiological range (≤10 μM), phytol and its metabolites can be cytotoxic to various cancer cell lines (Idel *et al.*, 2002; Mobley *et al.*, 2003; Kahlert *et al.*, 2005; Reiser *et al.*, 2006; Tang *et al.*, 2007; Rönicke *et al.*, 2009; Che *et al.*, 2013; Pejin *et al.*, 2014; Kim *et al.*, 2015; Nagai, 2015; Thakor *et al.*, 2017), as shown in Table 2. Although the induction period and measures of cytotoxicity slightly vary among studies, the lowest concentrations at which cytotoxicity was observed were 5 µM of PA in neuroblastoma Neuro2a cells (Nagai, 2015) and 8.8 µM of phytol in breast adenocarcinoma MCF-7 (Pejin *et al.*, 2014). The highest concentration at which cytotoxicity was observed was 79 µM of phytol in prostate carcinoma PC-3 (Pejin *et al.*, 2014), suggesting that cancer cell lines may differ in their sensitivity to phytol and its metabolites. Of note is that, in a previous study of lung cancer A549 cells, the IC_50_ for phytol (17 μM) was lower than for the cancer drug, methotrexate (20 µM) (Thakor *et al.*, 2017). Of concern is that 10 µM of PA was already cytotoxic to a human normal prostate epithelial cell line (NPrEC), but not to lymph node prostate carcinoma cells (Mobley *et al.*, 2003), suggesting a narrow and sometimes overlapping range between beneficial and detrimental effects. Additional studies are needed to investigate cytotoxicity of phytol and its metabolites in non-cancer and cancer cells and to elucidate why cell lines from different organs vary in their sensitivity to phytol and its metabolites.

**Table 2:**
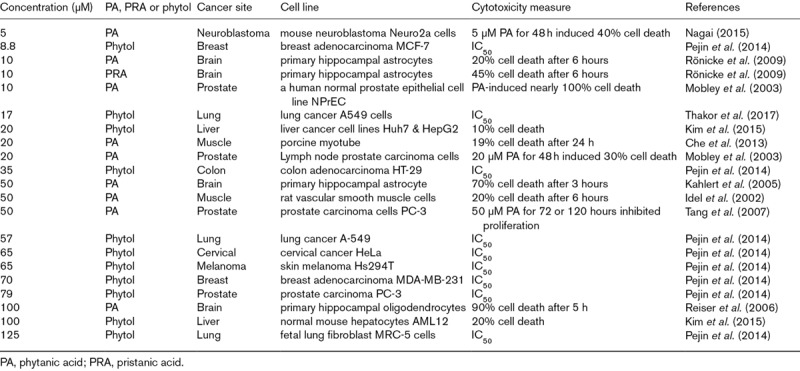
Effect of phytol, phytanic acid or pristanic acid on cytotoxicity in cell culture studies

Another desirable aspect of phytol and its metabolites as potential pharmacological agents is that they may improve markers of metabolic syndrome in the physiological range (≤10 μM). Organs may differ in their sensitivity to phytol and its metabolites, as minimal concentrations required for beneficial effects varied from 0.1 and 100 μM of PA. At concentration of as low as 0.1 μM, PA increased brown adipocyte differentiation (Schluter *et al.*, 2002), whereas 40 or 100 μM was needed for white adipocyte differentiation (Heim *et al.*, 2002; Schluter *et al.*, 2002). In muscle cells, 1 μM of PA was sufficient to stimulate glucose uptake (Che *et al.*, 2013) and 5 μM of phytol was sufficient to induce genes involved in muscle differentiation in C2C12 cells (Yang *et al.*, 2017). In contrast, 100 μM of PA was needed to increase transcription of genes that increase glucose uptake in HepG2 cells (Heim *et al.*, 2002).

**Fig. 2:**
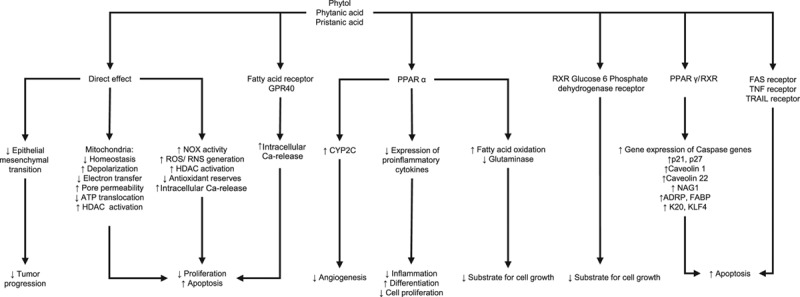
Proposed mechanisms by which phytol and its metabolites exert their chemo-preventive properties. Phytol, phytanic acid and pristanic acid are proposed to affect pathways involved in carcinogenesis such as cell proliferation, differentiation and apoptosis, angiogenesis and inflammation. These effects are direct or indirect by inducing activation of transcription factors such as PPAR and receptors related to fatty acid and energy metabolism or inflammation. PPAR, peroxisome proliferator-activated receptor.

Proposed pathways by which phytol and its metabolites exert their chemopreventive effects are summarized in Fig. 2. The primary proposed mode of action is that phytol and its metabolites act as natural ligands of various nuclear receptors, especially PPAR-α and -γ and RXR, and induce transcription of nuclear receptor-responsive genes. Such induction can occur at concentrations similar to synthetic ligands that can be used for insulin-sensitizing effects and chemoprevention and treatment (Peters *et al.*, 2012). Phytol concentrations as low as 1, 50 and 10 μM induced PPAR-α, -β and -γ activity, respectively, in monkey kidney CV-1 cells and HepG2 hepatocytes (Heim *et al.*, 2002; Goto *et al.*, 2005). Concentrations of 1 μM of PRA and 3 μM of PA induced RXR activity, potentially through retinol esters of PA (Tang *et al.*, 2007), in monkey kidney COS-1 cells and human HepG2 cells (Zomer *et al.*, 2000). Other proposed pathways are the PI3kinase/AKT pathway (Wang *et al.*, 2017) and as ligands of TRAIL, FAS, and TNF receptors, glucose-6-phosphate dehydrogenase receptor (Thakor *et al.*, 2017), and the free fatty acid receptor, GPR40 (Kruska and Reiser, 2011).

In summary, it is encouraging that phytol and its metabolites can alter pathways involved in carcinogenesis at concentrations within the physiological range (≤10 μM). It is, however, concerning that limited evidence also suggests that phytol or PA at the same concentrations can be cytotoxic to breast and brain non-cancer cell lines and that the chemo-preventive effect of phytol and its metabolites may differ by organs and cancer cell lines.

#### Animal model studies

Current evidence from animal models on a role of phytol and its metabolites in carcinogenesis is limited to one study (Liska *et al.*, 2012); hence, this review includes animal models of metabolic syndrome and nonalcoholic fatty liver disease (NAFLD) and animal toxicity studies (Ellinghaus *et al.*, 1999; Elmazar and Nau, 2004; Mönnig *et al.*, 2004; Hashimoto *et al.*, 2006; Mackie *et al.*, 2009; Elmazar *et al.*, 2013; Silva *et al.*, 2014; Selkälä *et al.*, 2015; Wang *et al.*, 2017; Yang *et al.*, 2017) (Table 3). Dietary concentrations of phytol and its metabolites are expressed in wt%, if possible, to facilitate comparison of dietary concentrations among species. In female Sprague–Dawley rats, the combination of phytol at 0.05 wt% and a vitamin D analogue seocalcitol at 7 ppb, starting 7 weeks after mammary tumor induction, slowed mammary tumor growth, but not tumor malignancy or invasiveness; (Liska *et al.*, 2012). Four animal feeding studies evaluated the effect of oral phytol on indicators of metabolic syndrome and NAFLD and showed beneficial effects (van den Brink *et al.*, 2005; Hashimoto *et al.*, 2006; Elmazar *et al.*, 2013; Wang *et al.*, 2017). Dietary concentrations ranged from 0.025 to 0.5 wt% of phytol for 2–5 weeks and resulted in decrease in body fat percentage and TNF-α concentrations, and improvement in blood lipid and glucose profiles (Hashimoto *et al.*, 2006; Elmazar *et al.*, 2013). Interestingly, in one animal study, the 0.05 wt% phytol increased adipogenesis, but decreased total body fat mass, as the average size of adipocytes was smaller (Wang *et al.*, 2017). In addition to indicators of metabolic syndrome and NAFLD, phytol also showed anti-inflammatory properties in two studies, specifically decreased concentrations of TNF-α in one study (Elmazar *et al.*, 2013) and decreased cytokine concentrations and inhibiting leukocyte migration in the other study (Silva *et al.*, 2014).

**Table 3:**
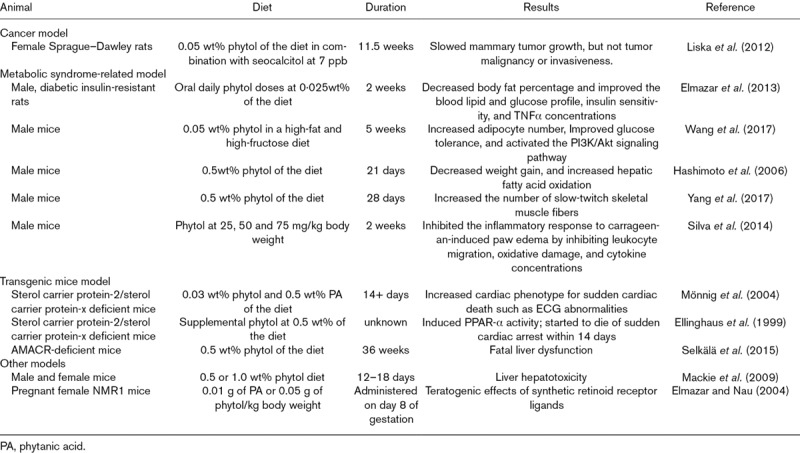
Effects of phytol, or phytanic acid on cancer or biomarkers in animal feeding studies

Detrimental effects of oral phytol and its metabolites were reported in four animal studies, three of which used transgenic animal models that cannot catabolize PA, which accumulates in tissues and subsequently leads to death. In two studies of sterol carrier protein-2/sterol carrier protein-x deficient mice, phytol at 0.5 wt% of the diet-induced PPAR-α activity (Ellinghaus *et al.*, 1999), and mice started to die of sudden cardiac arrest by two weeks (Mönnig *et al.*, 2004). In AMACR-deficient mice, phytol at 0.5 wt% of the diet resulted in morbidity by 2 weeks, killed most mice by 6 weeks, and all by 36 weeks due to liver dysfunction (Selkälä *et al.*, 2015). Hepatotoxicity, consistent with chronic PPAR-α upregulation, was also observed in male and female mice after being on a 0.5 or 1.0 wt% phytol diet for 12 days (Mackie *et al.*, 2009). Not based on transgenic animal model, but in pregnant NMR1 mice, teratogenic effects of synthetic retinoid receptor ligands were potentiated with oral administration of 0.01 g of PA or 0.05 g of phytol/kg of body weight (Elmazar and Nau, 2004).

In summary, results of oral phytol at concentrations of 0.05 wt% of the diet for prevention or treatment of metabolic syndrome and inflammation are encouraging; however, 10-fold higher concentrations may be harmful, indicating the need for dose-response studies. Given that a Western diet (assuming 500 g/day of macronutrient consumption) contains phytol at 10% of the PA intake, an average person would consume 5 mg/day (equal to 0.001 wt% of the diet) and 20 mg/day of phytol (0.004 wt% of the diet), which is 10-fold lower than the lowest concentrations shown effective in animal models (Elmazar *et al.*, 2013; Wang *et al.*, 2017). Furthermore, future animal studies should use dietary PA and PRA rather than phytol because humans have limited ability to metabolize phytol to PA or PRA and biologically effective PA and PRA contents can be obtained from human diet, assuming that phytol and its metabolites have the same efficacy (Steinberg, 1989; Brown *et al.*, 1993).

#### Human studies

Current evidence from intervention studies on a role of phytol and its metabolites is extremely limited. As to PA, initially, Werner *et al.* (2011) examined the effect of PA on biomarkers of metabolic syndrome among 14 healthy participants in a double-blind, randomized, 4-week parallel intervention study in Denmark. They were given 45 g of milk fat/day from test butter and cheese with 0.13 wt% of PA for the control group or 0.24 wt% of PA for the intervention group. Plasma PA concentrations increased in both groups significantly (P < 0.05) with a tendency of larger changes in the control group than the intervention group and no group differences in biomarkers of metabolic syndrome. In the follow-up study, Werner *et al.* (2013) increased both the PA amount and the length of intervention and compared the effect of replacing 15% of the participants’ caloric intake from fat in the form of butter (39 g/day of butter that included 156 vs. 78 mg/day of PA) for 12 weeks on blood lipid, glucose, and inflammatory marker profiles among 38 healthy participants. To standardize the PA content and minimize residual confounding, the milk fat was obtained either from pasture-grazing or conventionally-fed cows and the former had twice the PA concentrations. Overall, there were no significant group differences in biomarker profiles related to metabolic syndrome (Werner *et al.*, 2013). This null result can be explained by no meaningful difference in plasma PA concentrations (3.6 vs. 3.3 µM; *P* = 0.15), as only 15% of caloric intake from fat was replaced. Hence, future dietary intervention studies need to increase the difference in the PA content between two groups to achieve at least 2-fold differences in circulating PA concentration, potentially by providing the entire meals, instead of partially replacing their food source of fat. In short, there is currently lack of evidence from human intervention studies that would suggest a beneficial effect of phytol and its metabolites on chronic disease prevention.

Current evidence from epidemiological studies on a role of phytol and its metabolites in chronic diseases is limited to five cancer studies. Two studies (Wright *et al.*, 2012; Ollberding *et al.*, 2013) have examined the association between dietary PA intake and cancer risk. A retrospective case-control study (336 cases with 185 women and 460 controls with 236 women) in Nebraska, NLS (Ollberding *et al.*, 2013), reported no association between total dietary PA intake and NHL risk. When stratified by NHL subtype, total dietary PA was positively associated with follicular lymphoma and small lymphocytic lymphoma/chronic lymphocytic leukemia. Interestingly, the association between PA and NHL subtype differed by dietary PA source (Ollberding *et al.*, 2013). PA from beef, but not dairy products, was positively associated with diffuse large B-cell lymphoma. PA from milk, but not other dairy products, was associated with follicular lymphoma. This observation makes it unlikely that PA was driving the association with NHL subtype risk, leaving the possibility of residual confounding by other compounds in these food sources of PA. In the prospective ATBC cohort (27,111 participants, 1,929 incident cases, 438 advanced cases), PA intake was not associated with overall prostate cancer risk, but was positively associated with advanced prostate risk (Wright *et al.*, 2012). Moreover, this association was linked to high-fat dairy products and there was no association with PA from beef or fish. Thus, the association is subject to residual confounding as in NLS and could be due to compounds unique to dairy fat, such as short- and medium-chain fatty acids, which warrant further investigations. However, both studies did not further elucidate this possibility by mutually adjusting for the intake of these compounds, which needs to be explored in future studies and would help to clarify which bioactive compounds in dairy and beef products (e.g., mutagens) are driving the association between PA intake and cancer risk.

Three epidemiological studies measured serum or plasma PA concentrations and assessed the association with prostate cancer risk in men (Xu *et al.*, 2005; Price *et al.*, 2010; Wright *et al.*, 2014). A small, population-based, retrospective case-control study (49 cases and 55 control) conducted in North Carolina reported significantly higher serum PA levels among prostate cancer cases than controls (Xu *et al.*, 2005). Two prospective case-control studies nested within the European Prospective Investigation into Cancer and Nutrition (EPIC) and ATBC cohorts followed up on the finding. In the EPIC cohort (566 cases and 566 controls), plasma PA concentrations were not associated with overall prostate cancer risk; however, there was a positive association among participants who fasted at least three hours before blood draw (Price *et al.*, 2010). In the ATBC cohort (300 cases and 300 controls), in which dietary PA intake was associated with aggressive, but not overall prostate cancer risk (Wright *et al.*, 2012), neither serum PA or PRA concentrations were associated with overall or aggressive prostate cancer risk (Wright *et al.*, 2014).

As a note of caution, circulating PA concentrations do not necessarily reflect tissue PA concentrations. The correlation between serum and benign prostate tissue PA concentrations in 26 participants from Chicago was low (*r* = 0.28; *P* = 0.15) (Kataria *et al.*, 2015). More research is needed to determine factors that alter tissue PA concentrations and their relation with cancer risk and circulating PA concentrations. Besides prostate cancer, future epidemiologic studies are warranted to investigate cancer sites that have a lower IC_50_, such as breast or brain, or that accumulate more PA and have a higher lipid content, specifically liver and kidney (Hansen, 1965; Skrbic and Cumings, 1969; Malmendier *et al.*, 1974), which are more likely to show an association.

In summary, current evidence in human studies for a role of phytol and its metabolites in cancer prevention is insufficient, inconsistent, and could be explained by residual confounding by other food components. Another concern is that primary food sources of phytol and its metabolites have other food components that are known to have cancer-promoting properties (e.g. mutagens and iron in meats) (World Cancer Research Fund and American Institute for Cancer Research, 2018). The question arises whether dietary intervention studies for cancer prevention can be formulated to achieve large differences in circulating concentrations of phytol, its metabolites, or both and their chemo-preventive effects without residual confounding by other food components. Thus, a better strategy for future human intervention studies would be to use a supplement rich in phytol and its metabolites. As to cancer site, we recommend that future epidemiological studies should also examine cancers other than prostate cancer and NHL, especially cancer sites whose cells are known to be more sensitive to phytol and its metabolites such as brain, liver, kidney and blood. Furthermore, future studies should examine the interrelation between dietary, circulating and tissue levels of phytol and its metabolites in normal and cancer tissue.

### Role of phytol and its metabolites on α-methylacyl-CoA racemase expression and cancer risk

The enzyme AMACR is critical for catabolism of PA and PRA, as it converts the (R)-form of PRA to the (S)-form, which can be further oxidized in humans. The expression of AMACR is upregulated *in vitro* by PA or PRA (Mobley *et al.*, 2003) via PPAR-α to prevent accumulation of PA or PRA (Gloerich *et al.*, 2007). Thus, elevated PA levels in circulation may be linked to decreased AMACR expression and may differ by *AMACR* polymorphisms. There is continued interest in the association of AMACR expression and polymorphisms with cancer, as elevated AMACR protein or gene overexpression has been reported in various cancer tissues such as prostate, colon, rectum, ovaries, breast, bladder, lung, kidney, skin and lymphoma (Zhou *et al.*, 2002). Currently, *AMACR* gene expression is used in prostate cancer diagnosis in combination with other biomarkers such as 34E12, CK5/6 or p63 (Amin *et al.*, 2014) or with prostate cancer antigen 3 (Ouyang *et al.*, 2009), as AMACR is most consistently upregulated in prostate cancer tissue (Thornburg *et al.*, 2006). A meta-analysis combining five case-control studies found that *D175G* and *M9V* polymorphisms of the *AMACR* gene were associated with prostate cancer risk (Chen *et al.*, 2015).

A study conducted in Chicago using benign prostate tissue did not find an association between *AMACR* gene expression and dietary PA intake or serum PA concentration (Kataria *et al.*, 2015). Furthermore, *AMACR* polymorphism did not modify the associations between dietary red meat or dairy intake on prostate cancer in the population-based case-control study in the state of Washington (Wright *et al.*, 2011). AMACR is generally required to convert (R)-forms of fatty acids with chiral centers (usually methylated fatty acids of microbial origin) to (S)-forms of fatty acids and, thus, are essential for the degradation of bile acids, ibuprofen and other methylated fatty acids (Wanders, 2014). Therefore, elevated AMACR expression may be due to PPAR-α stimulation by compounds other than phytol or its metabolites, such as increased bile acid turnover, increased microbial fatty acid degradation, and low activity of AMACR or enzymes downstream required for methylated fatty acid degradation. Future research is needed to determine factors that elevate AMACR expression in some cancer tissues, but not in others. In summary, while the association of *AMACR* gene and protein expression with cancer risk and prognosis is well-established for prostate cancer, there is limited evidence that this association is modified by PA.

## Conclusion

There is a gap in research and knowledge related to phytol and its metabolites, PA and PRA and carcinogenesis. Phytol and its metabolites are potential dietary compounds for cancer prevention because, based on some evidence in cell culture studies and limited evidence in animal models, they can alter pathways involved in carcinogenesis at physiological concentrations that can be achieved by dietary modifications. However, there are concerns regarding the cytotoxicity in non-cancer cells and various cell types. More research is needed to determine if and how normal and cancer tissues react similarly and differently to phytol and its metabolites. Animal feeding studies at phytol concentrations that can be achieved with dietary modification alone show promise; however, the number of studies is insufficient to draw conclusions. Moreover, studies in transgenic animal models report higher morbidity and mortality if PA accumulates in tissues. Previous studies on circulating and tissue PA and PRA concentrations suggest that little is known about the physiological processes involved in the control of PA and PRA concentrations in absorption, circulation and tissue distribution as well as the effect of the intestinal microbiome on circulating PA and PRA concentrations. Population-based studies that examine the association between phytol and its metabolites and cancer risk are few and largely limited to prostate cancer. Based on the cytotoxicity and tissue accumulation data, other cancer sites such as brain, liver, kidney and blood are more sensitive to PA and, thus, have a greater chance to show associations in epidemiological studies than other cancer sites. In conclusion, phytol and its metabolites are potential natural agents for cancer prevention if current challenges including cytotoxicity in non-cancer cells and morbidity and mortality associated with PA accumulation in tissue can be mitigated.

## Acknowledgements

This study was supported by the Knight Cancer Institute OHSU/OSU Cancer Prevention and Control Initiative (PHR030-PV07 to Y.T.). The funders had no role in the submission of the manuscript. We thank Ms. Alexandra Heisler and Dr. Jessica Keune for their assistance in editing the manuscript.

G.B. and Y.T. was responsible for the conceptualization of this review. G.B., Z.Z. and Y.T. conducted literature search and G.B. drafted the initial manuscript. R.K., M.G., J.S. and Y.T. provided critical comments to the manuscript. All authors reviewed, revised the manuscript and approved the final version.

## Conflicts of Interest

There are no conflicts of interest.

## References

[R1] Allen NE Grace PB Ginn A Travis RC Roddam AW Appleby PN Key T Phytanic acid: measurement of plasma concentrations by gas-liquid chromatography-mass spectrometry analysis and associations with diet and other plasma fatty acids. Br J Nutr. 2008; 99:653–659 17868488 10.1017/S000711450782407X

[R2] Amin MB Epstein JI Ulbright TM Humphrey PA Egevad L Montironi R Best practices recommendations in the application of immunohistochemistry in urologic pathology: report from the International Society of Urological Pathology consensus conference. Am J Surg Pathol. 2014; 38:1017–1022 25025364 10.1097/PAS.0000000000000254

[R3] Siegel RL Miller KD Jemal A Cancer statistics, 2018. CA Cancer J Clin. 2018; 68:7–30 29313949 10.3322/caac.21442

[R4] Baldwin EJ Gibberd FB Harley C Sidey MC Feher MD Wierzbicki AS The effectiveness of long-term dietary therapy in the treatment of adult Refsum disease. J Neurol Neurosurg Psychiatry. 2010; 81:954–957 20547622 10.1136/jnnp.2008.161059

[R5] Baxter JH Absorption of chlorophyll phytol in normal man and in patients with Refsum’s disease. J Lipid Res. 1968; 9:636–641 4177872

[R6] Borges CG Canani CR Fernandes CG Zanatta Â Seminotti B Ribeiro CA Reactive nitrogen species mediate oxidative stress and astrogliosis provoked by in vivo administration of phytanic acid in cerebellum of adolescent rats: a potential contributing pathomechanism of cerebellar injury in peroxisomal disorders. Neuroscience. 2015; 304:122–132 26188285 10.1016/j.neuroscience.2015.07.028

[R7] Brown PJ Mei G Gibberd FB Burston D Mayne PD Mcclinchy JE Diet and Refsums disease - the determination of phytanic acid and phytol in certain foods and the application of this knowledge to the choice of suitable convenience foods for patients with Refsums disease. J Hum Nutr Diet. 1993; 6:295–305

[R8] Che BN Oksbjerg N Hellgren LI Nielsen JH Young JF Phytanic acid stimulates glucose uptake in a model of skeletal muscles, the primary porcine myotubes. Lipids Health Dis. 2013; 12:14 23398851 10.1186/1476-511X-12-14 PMC3606424

[R9] Chen N Wang JR Huang L Yang Y Jiang YM Guo XJ Significant association of alpha-methylacyl-coa racemase gene polymorphisms with susceptibility to prostate cancer: a meta-analysis. Asian Pac J Cancer Prev. 2015; 16:1857–1863 25773837 10.7314/apjcp.2015.16.5.1857

[R10] Coppack SW Evans R Gibberd FB Clemens ME Billimoria JD Can patients with Refsum’s disease safely eat green vegetables?. Br Med J (Clin Res Ed). 1988; 296:828 10.1136/bmj.296.6625.828 PMC2545156 2453246

[R11] Dhaunsi GS Alsaeid M Akhtar S Phytanic acid activates NADPH oxidase through transactivation of epidermal growth factor receptor in vascular smooth muscle cells. Lipids Health Dis. 2016; 15:105 27287039 10.1186/s12944-016-0273-9 PMC4902935

[R12] Dhaunsi GS Alsaeid M Akhtar S Phytanic acid attenuates insulin-like growth factor-1 activity via nitric oxide-mediated γ-secretase activation in rat aortic smooth muscle cells: possible implications for pathogenesis of infantile Refsum disease. Pediatr Res. 2017; 81:531–536 27886192 10.1038/pr.2016.258

[R13] Ellinghaus P Wolfrum C Assmann G Spener F Seedorf U Phytanic acid activates the peroxisome proliferator-activated receptor alpha (pparalpha) in sterol carrier protein 2-/ sterol carrier protein x-deficient mice. J Biol Chem. 1999; 274:2766–2772 9915808 10.1074/jbc.274.5.2766

[R14] Elmazar MM El-Abhar HS Schaalan MF Farag NA Phytol/phytanic acid and insulin resistance: potential role of phytanic acid proven by docking simulation and modulation of biochemical alterations. PLoS One. 2013; 8:e45638 23300941 10.1371/journal.pone.0045638 PMC3534692

[R15] Elmazar MM Nau H Potentiation of the teratogenic effects induced by coadministration of retinoic acid or phytanic acid/phytol with synthetic retinoid receptor ligands. Arch Toxicol. 2004; 78:660–668 15558240 10.1007/s00204-004-0586-8

[R16] Gloerich J van den Brink DM Ruiter JP van Vlies N Vaz FM Wanders RJ Ferdinandusse S Metabolism of phytol to phytanic acid in the mouse, and the role of pparalpha in its regulation. J Lipid Res. 2007; 48:77–85 17015885 10.1194/jlr.M600050-JLR200

[R17] Goto T Takahashi N Kato S Egawa K Ebisu S Moriyama T Phytol directly activates peroxisome proliferator-activated receptor alpha (pparalpha) and regulates gene expression involved in lipid metabolism in pparalpha-expressing hepg2 hepatocytes. Biochem Biophys Res Commun. 2005; 337:440–445 16202384 10.1016/j.bbrc.2005.09.077

[R18] Grings M Tonin AM Knebel LA Zanatta A Moura AP Filho CS Phytanic acid disturbs mitochondrial homeostasis in heart of young rats: a possible pathomechanism of cardiomyopathy in Refsum disease. Mol Cell Biochem. 2012; 366:335–343 22527938 10.1007/s11010-012-1311-1

[R19] Gu H Huang T Shen Y Liu Y Zhou F Jin Y Reactive oxygen species-mediated tumor microenvironment transformation: the mechanism of radioresistant gastric cancer. Oxid Med Cell Longev. 2018; 2018:5801209 29770167 10.1155/2018/5801209 PMC5892229

[R20] Hansen RP 3,7,11,15-tetramethylhexadecanoic acid: its occurrence in the tissues of humans afflicted with Refsum’s syndrome. Biochim Biophys Acta. 1965; 106:304–310 4160183 10.1016/0005-2760(65)90038-x

[R21] Hansen RP Phytol - its metabolic products and their distribution - a review. New Zeal J Sci. 1980; 23:259–275

[R22] Hashimoto T Shimizu N Kimura T Takahashi Y Ide T Polyunsaturated fats attenuate the dietary phytol-induced increase in hepatic fatty acid oxidation in mice. J Nutr. 2006; 136:882–886 16549445 10.1093/jn/136.4.882

[R23] Heim M Johnson J Boess F Bendik I Weber P Hunziker W Fluhmann B Phytanic acid, a natural peroxisome proliferator-activated receptor (PPAR) agonist, regulates glucose metabolism in rat primary hepatocytes. FASEB J. 2002; 16:718–720 11923221 10.1096/fj.01-0816fje

[R24] Idel S Ellinghaus P Wolfrum C Nofer JR Gloerich J Assmann G Branched chain fatty acids induce nitric oxide-dependent apoptosis in vascular smooth muscle cells. J Biol Chem. 2002; 277:49319–49325 12368296 10.1074/jbc.M204639200

[R25] Islam MT de Alencar MV da Conceição Machado K da Conceição Machado K de Carvalho Melo-Cavalcante AA de Sousa DP de Freitas RM Phytol in a pharma-medico-stance. Chem Biol Interact. 2015; 240:60–73 26296761 10.1016/j.cbi.2015.07.010

[R26] Kahlert S Schönfeld P Reiser G The Refsum disease marker phytanic acid, a branched chain fatty acid, affects ca2+ homeostasis and mitochondria, and reduces cell viability in rat hippocampal astrocytes. Neurobiol Dis. 2005; 18:110–118 15649701 10.1016/j.nbd.2004.08.010

[R27] Kataria Y Wright M Deaton RJ Rueter EE Rybicki BA Moser AB Dietary influences on tissue concentrations of phytanic acid and AMACR expression in the benign human prostate. Prostate. 2015; 75:200–210 25307752 10.1002/pros.22905 PMC4778716

[R28] Kim CW Lee HJ Jung JH Kim YH Jung DB Sohn EJ Activation of caspase-9/3 and inhibition of epithelial mesenchymal transition are critically involved in antitumor effect of phytol in hepatocellular carcinoma cells. Phytother Res. 2015; 29:1026–1031 25892665 10.1002/ptr.5342

[R29] Komiya T Kyohkon M Ohwaki S Eto J Katsuzaki H Imai K Phytol induces programmed cell death in human lymphoid leukemia molt 4B cells. Int J Mol Med. 1999; 4:377–380 10493978 10.3892/ijmm.4.4.377

[R30] Kruska N Reiser G Phytanic acid and pristanic acid, branched-chain fatty acids associated with Refsum disease and other inherited peroxisomal disorders, mediate intracellular ca2+ signaling through activation of free fatty acid receptor GPR40. Neurobiol Dis. 2011; 43:465–472 21570468 10.1016/j.nbd.2011.04.020

[R31] Leipnitz G Amaral AU Zanatta A Seminotti B Fernandes CG Knebel LA Neurochemical evidence that phytanic acid induces oxidative damage and reduces the antioxidant defenses in cerebellum and cerebral cortex of rats. Life Sci. 2010; 87:275–280 20619275 10.1016/j.lfs.2010.06.015

[R32] Liska J Macejova D Ondkova S Brtko J Morphology of 1-methyl-1-nitrosourea induced rat mammary tumours after treatment with precursor of phytanic acid or its combination with vitamin D analogue. Endocr Regul. 2012; 46:21–26 22329818 10.4149/endo_2012_021

[R33] Lloyd MD Darley DJ Wierzbicki AS Threadgill MD Alpha-methylacyl-coa racemase–an ‘obscure’ metabolic enzyme takes centre stage. FEBS J. 2008; 275:1089–1102 18279392 10.1111/j.1742-4658.2008.06290.x

[R34] Lloyd MD Yevglevskis M Lee GL Wood PJ Threadgill MD Woodman TJ Α-methylacyl-coa racemase (AMACR): metabolic enzyme, drug metabolizer and cancer marker P504S. Prog Lipid Res. 2013; 52:220–230 23376124 10.1016/j.plipres.2013.01.001

[R35] Mackie JT Atshaves BP Payne HR McIntosh AL Schroeder F Kier AB Phytol-induced hepatotoxicity in mice. Toxicol Pathol. 2009; 37:201–208 19188468 10.1177/0192623308330789 PMC2838495

[R36] Malmendier CL Jonniaux G Voet W Van Den Bergen CJ Fatty acid composition of tissues in Refsum’s disease (herodopathia atactica polyneuritiformis). Estimation of total phytanic acid accumulation. Biomedicine. 1974; 20:398–407 4141904

[R37] Mobley JA Leav I Zielie P Wotkowitz C Evans J Lam YW Branched fatty acids in dairy and beef products markedly enhance alpha-methylacyl-coa racemase expression in prostate cancer cells in vitro. Cancer Epidemiol Biomarkers Prev. 2003; 12:775–783 12917210

[R38] Mönnig G Wiekowski J Kirchhof P Stypmann J Plenz G Fabritz L Phytanic acid accumulation is associated with conduction delay and sudden cardiac death in sterol carrier protein-2/sterol carrier protein-x deficient mice. J Cardiovasc Electrophysiol. 2004; 15:1310–1316 15574183 10.1046/j.1540-8167.2004.03679.x

[R39] Nagai K Phytanic acid induces neuro2a cell death via histone deacetylase activation and mitochondrial dysfunction. Neurotoxicol Teratol. 2015; 48:33–39 25619426 10.1016/j.ntt.2015.01.006

[R40] Ollberding NJ Aschebrook-Kilfoy B Caces DB Wright ME Weisenburger DD Smith SM Chiu BC Phytanic acid and the risk of non-hodgkin lymphoma. Carcinogenesis. 2013; 34:170–175 23042099 10.1093/carcin/bgs315 PMC3534193

[R41] Ouyang B Bracken B Burke B Chung E Liang J Ho SM A duplex quantitative polymerase chain reaction assay based on quantification of alpha-methylacyl-coa racemase transcripts and prostate cancer antigen 3 in urine sediments improved diagnostic accuracy for prostate cancer. J Urol. 2009; 181:2508–2513; discussion 2513 19371911 10.1016/j.juro.2009.01.110 PMC4372725

[R42] Pejin B Kojic V Bogdanovic G An insight into the cytotoxic activity of phytol at in vitro conditions. Nat Prod Res. 2014; 28:2053–2056 24896297 10.1080/14786419.2014.921686

[R43] Peters JM Shah YM Gonzalez FJ The role of peroxisome proliferator-activated receptors in carcinogenesis and chemoprevention. Nat Rev Cancer. 2012; 12:181–195 22318237 10.1038/nrc3214 PMC3322353

[R44] Price AJ Allen NE Appleby PN Crowe FL Jenab M Rinaldi S Plasma phytanic acid concentration and risk of prostate cancer: results from the European Prospective Investigation into Cancer and Nutrition. Am J Clin Nutr. 2010; 91:1769–1776 20427733 10.3945/ajcn.2009.28831 PMC5749610

[R45] Reiser G Schönfeld P Kahlert S Mechanism of toxicity of the branched-chain fatty acid phytanic acid, a marker of Refsum disease, in astrocytes involves mitochondrial impairment. Int J Dev Neurosci. 2006; 24:113–122 16386870 10.1016/j.ijdevneu.2005.11.002

[R46] Roca-Saavedra P Mariño-Lorenzo P Miranda JM Porto-Arias JJ Lamas A Vazquez BI Phytanic acid consumption and human health, risks, benefits and future trends: a review. Food Chem. 2017; 221:237–247 27979198 10.1016/j.foodchem.2016.10.074

[R47] Rönicke S Kruska N Kahlert S Reiser G The influence of the branched-chain fatty acids pristanic acid and Refsum disease-associated phytanic acid on mitochondrial functions and calcium regulation of hippocampal neurons, astrocytes, and oligodendrocytes. Neurobiol Dis. 2009; 36:401–410 19703563 10.1016/j.nbd.2009.08.005

[R48] Samimi A Kalantari H Lorestani MZ Shirzad R Saki N Oxidative stress in normal hematopoietic stem cells and leukemia. Apmis. 2018; 126:284–294 29575200 10.1111/apm.12822

[R49] Schluter A Giralt M Iglesias R Villarroya F Phytanic acid, but not pristanic acid, mediates the positive effects of phytol derivatives on brown adipocyte differentiation. FEBS Lett. 2002; 517:83–86 12062414 10.1016/s0014-5793(02)02583-8

[R50] Schönfeld P Kahlert S Reiser G In brain mitochondria the branched-chain fatty acid phytanic acid impairs energy transduction and sensitizes for permeability transition. Biochem J. 2004; 383:121–128 15198638 10.1042/BJ20040583 PMC1134050

[R51] Schönfeld P Kahlert S Reiser G A study of the cytotoxicity of branched-chain phytanic acid with mitochondria and rat brain astrocytes. Exp Gerontol. 2006; 41:688–696 16616447 10.1016/j.exger.2006.02.013

[R52] Schönfeld P Reiser G Rotenone-like action of the branched-chain phytanic acid induces oxidative stress in mitochondria. J Biol Chem. 2006; 281:7136–7142 16410242 10.1074/jbc.M513198200

[R53] Schönfeld P Wojtczak L Fatty acids decrease mitochondrial generation of reactive oxygen species at the reverse electron transport but increase it at the forward transport. Biochim Biophys Acta. 2007; 1767:1032–1040 17588527 10.1016/j.bbabio.2007.04.005

[R54] Selkälä EM Nair RR Schmitz W Kvist AP Baes M Hiltunen JK Autio KJ Phytol is lethal for AMACR-deficient mice. Biochim Biophys Acta. 2015; 1851:1394–1405 26248199 10.1016/j.bbalip.2015.07.008

[R55] Silva RO Sousa FB Damasceno SR Carvalho NS Silva VG Oliveira FR Phytol, a diterpene alcohol, inhibits the inflammatory response by reducing cytokine production and oxidative stress. Fundam Clin Pharmacol. 2014; 28:455–464 24102680 10.1111/fcp.12049

[R56] Skrbic TR Cumings JN Phytanic acid in tissue lipids in Refsum’s disease. Clin Chim Acta. 1969; 23:17–21 4178397 10.1016/0009-8981(69)90004-7

[R57] Steinberg D Scriver CR Beaudet AA Sly WS Valle D Refsum disease. Metabolic Basis of Inherited Disease. 1989, New York, New York: McGraw Hill 1533–1550

[R58] Steinberg D Vroom FQ Engel WK Cammermeyer J Mize CE Avigan J Refsum’s disease--a recently characterized lipidosis involving the nervous system. Combined clinical staff conference at the National Institutes of Health. Ann Intern Med. 1967; 66:365–395 4163283 10.7326/0003-4819-66-2-365

[R59] Tang XH Suh MJ Li R Gudas LJ Cell proliferation inhibition and alterations in retinol esterification induced by phytanic acid and docosahexaenoic acid. J Lipid Res. 2007; 48:165–176 17068359 10.1194/jlr.M600419-JLR200

[R60] Thakor P Subramanian RB Thakkar SS Ray A Thakkar VR Phytol induces ROS mediated apoptosis by induction of caspase 9 and 3 through activation of TRAIL, FAS and TNF receptors and inhibits tumor progression factor glucose 6 phosphate dehydrogenase in lung carcinoma cell line (A549). Biomed Pharmacother. 2017; 92:491–500 28575806 10.1016/j.biopha.2017.05.066

[R61] Thornburg T Turner AR Chen YQ Vitolins M Chang B Xu J Phytanic acid, AMACR and prostate cancer risk. Future Oncol. 2006; 2:213–223 16563090 10.2217/14796694.2.2.213

[R62] van den Brink DM van Miert JN Dacremont G Rontani JF Wanders RJ Characterization of the final step in the conversion of phytol into phytanic acid. J Biol Chem. 2005; 280:26838–26844 15866875 10.1074/jbc.M501861200

[R63] Verhoeven NM Jakobs C Human metabolism of phytanic acid and pristanic acid. Prog Lipid Res. 2001; 40:453–466 11591435 10.1016/s0163-7827(01)00011-x

[R64] Vetter W Schroder M Concentrations of phytanic acid and pristanic acid are higher in organic than in conventional dairy products from the German market. Food Chem. 2010; 119:746–752

[R65] Wanders RJ Metabolic functions of peroxisomes in health and disease. Biochimie. 2014; 98:36–44 24012550 10.1016/j.biochi.2013.08.022

[R66] Wang J Hu X Ai W Zhang F Yang K Wang L Phytol increases adipocyte number and glucose tolerance through activation of PI3K/akt signaling pathway in mice fed high-fat and high-fructose diet. Biochem Biophys Res Commun. 2017; 489:432–438 28571740 10.1016/j.bbrc.2017.05.160

[R67] Werner LB Hellgren LI Raff M Jensen SK Petersen RA Drachmann T Tholstrup T Effect of dairy fat on plasma phytanic acid in healthy volunteers–a randomized controlled study. Lipids Health Dis. 2011; 10:95 21663648 10.1186/1476-511X-10-95 PMC3127790

[R68] Werner LB Hellgren LI Raff M Jensen SK Petersen R A Drachmann T Tholstrup T Effects of butter from mountain-pasture grazing cows on risk markers of the metabolic syndrome compared with conventional danish butter: a randomized controlled study. Lipids Health Dis. 2013; 12:99 23842081 10.1186/1476-511X-12-99 PMC3720277

[R69] World Cancer Research Fund and American Institute for Cancer Research. Food, Nutrition, Physical Activity, and the Prevention of Cancer: A Global Perspective. 2018, Washington, DC.: American Association for Cancer Research

[R70] Wright ME Albanes D Moser AB Weinstein SJ Snyder K Männistö S Gann PH Serum phytanic and pristanic acid levels and prostate cancer risk in Finnish smokers. Cancer Med. 2014; 3:1562–1569 25132681 10.1002/cam4.319 PMC4298383

[R71] Wright ME Bowen P Virtamo J Albanes D Gann PH Estimated phytanic acid intake and prostate cancer risk: a prospective cohort study. Int J Cancer. 2012; 131:1396–1406 22120496 10.1002/ijc.27372 PMC4415360

[R72] Wright JL Neuhouser ML Lin DW Kwon EM Feng Z Ostrander EA AMACR polymorphisms, dietary intake of red meat and dairy and prostate cancer risk. Prostate. 2011; 71:498–506 20945498 10.1002/pros.21267 PMC3148811

[R73] Xu J Thornburg T Turner AR Vitolins M Case D Shadle J Serum levels of phytanic acid are associated with prostate cancer risk. Prostate. 2005; 63:209–214 15712232 10.1002/pros.20233

[R74] Yang K Wang L Zhou G Lin X Peng J Wang L Phytol promotes the formation of slow-twitch muscle fibers through PGC-1α/mirna but not mitochondria oxidation. J Agric Food Chem. 2017; 65:5916–5925 28654264 10.1021/acs.jafc.7b01048

[R75] Zhou M Chinnaiyan AM Kleer C G Lucas PC Rubin MA Alpha-methylacyl-coa racemase: a novel tumor marker over-expressed in several human cancers and their precursor lesions. Am J Surg Pathol. 2002; 26:926–931 12131161 10.1097/00000478-200207000-00012

[R76] Zomer AW Jansen GA Van Der Burg B Verhoeven NM Jakobs C Van Der Saag PT Phytanoyl-coa hydroxylase activity is induced by phytanic acid. Eur J Biochem. 2000; 267:4063–4067 10866807 10.1046/j.1432-1327.2000.01451.x

